# Ketamine in acute phase of severe traumatic brain injury “an old drug for new uses?”

**DOI:** 10.1186/s13054-020-03452-x

**Published:** 2021-01-06

**Authors:** Daniel Agustin Godoy, Rafael Badenes, Paolo Pelosi, Chiara Robba

**Affiliations:** 1 Neurointensive Care Unit, Sanatorio Pasteur, Catamarca, Argentina; 2 Intensive Care Unit, Hospital Carlos Malbran, Catamarca, Argentina; 3 Anesthesiology and Surgical-Trauma Intensive Care, University Clinic Hospital, Valencia, Spain; 4 Department of Surgery, University of Valencia, Valencia, Spain; 5 INCLIVA Research Medical Institute, Valencia, Valencia Spain; 6 Anesthesia and Intensive Care, San Martino Policlinico Hospital, IRCCS for Oncology and Neurosciences, Genoa, Italy; 7 Department of Surgical Sciences and Integrated Diagnostics, University of Genoa, Genoa, Italy

**Keywords:** Ketamine, Traumatic brain injury, Sedation, Agitation, Secondary insult, Cooperative sedation, Neuroprotection

## Abstract

Maintaining an adequate level of sedation and analgesia plays a key role in the management of traumatic brain injury (TBI). To date, it is unclear which drug or combination of drugs is most effective in achieving these goals. Ketamine is an agent with attractive pharmacological and pharmacokinetics characteristics. Current evidence shows that ketamine does not increase and may instead decrease intracranial pressure, and its safety profile makes it a reliable tool in the prehospital environment. In this point of view, we discuss different aspects of the use of ketamine in the acute phase of TBI, with its potential benefits and pitfalls.

## Introduction

In the acute phase of severe Traumatic Brain Injury (TBI) it is fundamental to prevent and avoid secondary insults that can further aggravate primary brain injury [1]. Implementation of targeted sedation and analgesia is a cornerstone of optimizing patient comfort and minimizing distress, which may lead to unintended consequences including delayed recovery from critical illness and slower weaning from mechanical ventilation. The use of structured approaches to sedation management, including guidelines, protocols, and algorithms can promote evidence-based care, reduce variation in clinical practice, and systematically reduce the likelihood of excessive or prolonged sedation [2].

In patients with TBI, sedation has both “general” indications, including control of anxiety, pain, discomfort, agitation, facilitation of mechanical ventilation, and neurological-specific indications, such as reduction in cerebral metabolic demand, and tolerance to cerebral ischaemia. Sedation also is an essential therapeutic component of intracranial pressure therapy, targeted temperature management and seizure control.

Several different classes of sedative agents are used in the management of patients with TBI [2, 3]. These agents are used at induction of anesthesia, to maintain sedation, to reduce elevated intracranial pressure, to terminate seizure activity and facilitate ventilation.

Controversies regarding the optimal sedation management persist, especially in critically ill patients with neurological injury, who were systematically excluded from large randomized studies [3]. As consequence, recent guidelines for the control of agitation, pain and delirium in critically ill patients do not include brain injured patients and the Brain Trauma Foundation guidelines do not recommend a specific agent or a combination of them due to the lack of studies and solid scientific evidence [3, 4].

Ketamine is a potent analgesic, with dissociative and sedative properties. Differently from other drugs, ketamine does not suppress respiratory activity or airway reflexes (except at very high doses) and may have potential neuroprotective effects, as well as a potential in decreasing seizures and non-convulsive epileptic activity [5, 6]. These properties make it an interesting agent when profound analgesia and sedation are required. In this context, it is our intention to contribute with our point of view in relation to certain questions raised regarding sedation and analgesia in severe TBI, focusing on the potential use of ketamine—alone or in combination with other sedatives—an “old drug for new uses.”

## Optimization of sedation and analgesia in severe TBI patients

The current trend in the management of the critically ill patient is to avoid and/or minimize sedation, while optimizing analgesia and comfort [3]. In the general intensive care unit (ICU) adult and pediatric populations, minimizing or avoiding sedation has shown to provide a better outcome, [3] with shortening of time in mechanical ventilation, reduced length of stay in intensive care unit and early mobilization, potentially reducing costs and improving outcome [3]. However, these principles cannot be completely applied to severe TBI patients, for several reasons [7]. First, cerebral pathophysiology and the flow/ metabolism coupling can be altered after brain injury [1, 2]. The biological response to injury (metabolic, hormonal and inflammatory) agitation, delirium and pain predispose to the development of systemic (hypoxemia, hypercapnia, hypocapnia, hyperglycemia, hyperthermia, arterial hypotension) or intracranial secondary insults (intracranial pressure increase, brain ischemia or hypoxia), all factors that have a well-recognized negative impact on final outcome [7] (Additional file 1: Figure S1). On the other hand, the need for mechanical ventilation and other invasive monitoring systems require synchrony and a certain state of stillness to facilitate their adaptation and targeting physiological parameters. Additionally, certain specific situations (intracranial hypertension, seizures, sympathetic hyperactivity, embolism) or therapeutic strategies (controlled normothermia) require sedation to optimize their control and management [7].

## What is the appropriate level of sedation and how to monitor it?

This is a debated, controversial and unclear aspect [8], as there are no specific guidelines or recommendations addressing this point in severe TBI [8]. However, in our opinion, it is important to establish certain objectives based on premises obtained from common sense and individual experience supported by the scientific knowledge that the available literature provides. Sedation and analgesia are dynamic processes that must meet specific goals, be controlled, and be easily modified according to the evolution of patient’s status. Knowledge on the pharmacokinetic and pharmacodynamic as well as the safety profile of the drugs to be used is essential to minimize their harmful effects. Planning a strategy, protocolizing and monitoring treatment are the pillars on which a good analgesia and sedation strategy should rely. These aspects contribute to achieving an optimal level of sedation aimed at balancing the deleterious effects of under or over sedation (Additional file 2: Figure S2). Regarding monitoring methods to assess sedation to be used at the bedside, to date, there is no evidence that supports one over the other [3]. Both clinical scales (Richmond Agitation–Sedation Scale RASS) or objective methods based on the electroencephalography (EEG) may provide helpful information [9, 10]. In general, it is recommended to initially maintain a RASS score of − 3, − 4 adjusting according to the previously stated therapeutic objectives [11]. However, possible contradictions and confusions may exist between use of RASS and Glasgow Coma Score in severe head trauma [12, 13].

The influence of ketamine on the EEG tracing is dose dependent. At the usual doses that induce unconsciousness, ketamine produces a pattern called “gamma burst” characterized by the alternation of slow delta waves (0.1–4 Hz) with gamma waves (27–40 Hz) associated with an increase in theta waves (4–8 Hz) and decrease in alpha and beta waves [14, 15].

Quantitative EEG, which calculates a time-compressed simplified visual display from raw EEG data, can be helpful for the early detection of over-sedation or brain injury worsening, and of non-convulsive states [11].

## Is ketamine an option for sedation in severe TBI?

Ketamine is primarily a competitive antagonist of *N*-methyl-d-aspartate receptors although it also acts on opioid (μ), and muscarinic receptors, and on sodium channels [11, 16–18]. It has analgesic, hypnotic, anesthetic and sympathomimetic properties. Its main action site is the thalamo-cortical system [11, 16–18]. This agent inhibits presynaptic reuptake of catecholamines, with an onset of 30 s, and then rapidly distributes to the central nervous system for being highly lipophilic (distribution half-life of 10 min) [11, 16–18]. It is then metabolized in the liver, generating active compounds (norketamine and hydroxynorketamine) and is eliminated almost entirely in the urine with an elimination half-life of 1.5 to 3 h [11, 16–21].

Because of an oxidation by a microsomal enzyme system (*N*‐demethylation), ketamine is mostly metabolized in nor-ketamine (80%), an active metabolite that is hydroxylized to hydroxy-nor-ketamine (15%), to be excreted in bile (3%) and urine (91%) after glucuronoconjugation [16–21].

Ketamine demethylation to norketamine, is a reaction that is catalyzed primarily by the cytochrome P450 complex and by liver enzymes CYP2B6 and CYP3A4. The individual variability in the metabolism of ketamine and the possible tolerance have been attributed, in part, to differences in the expression of P450 enzymes [19–21].

Enzimatic CYP2B6 and CYP3A4 inducers such as carbamazepine, phenobarbital, phenytoin, or rifampicin can reduce serum ketamine levels [19–21]. There are few data related to ketamine metabolism in conditions of kidney and liver failure. The influence of kidney function on ketamine pharmacokinetics is low. During renal dysfunction, ketamine concentrations are approximately 20% higher [19–21]. Experimentally, ketamine has very little influence on the arterial or portal liver blood flow. Since ketamine clearance is primarily hepatic in cases of severe insufficiency, it is recommended to reduce the dose to be used by half [19–21].

The usual dose used in the clinical practice ranges between 1 and 5 mg/kg/h [11, 16–18].

The target of employed doses of ketamine alone or in combination con other drugs should be directed to achieve the RASS or quantitative EEG goal.

## Which are the systemic effects of ketamine?

The respiratory drive and the protective reflexes of the upper airway are generally preserved after ketamine administration, thus allowing to maintain spontaneous ventilation [16–21]. However, for high doses that would serve an anesthetic effect or be used for seizure or spreading depolarization management (> 1 mg/kg) there is a risk of respiratory suppression and dissociation [16–21]. In particular, clinical data and case studies support a therapeutic effect of ketamine in suppression of spreading depolarization following brain injury. Preclinical data strongly support efficacy in terms of frequency of spreading depolarization (SD) as well as recovery from electrocorticography depression. This is fundamental as SD has been suggested as an important mechanism for secondary brain injury and delayed cerebral ischemia [22–24]. Ketamine also causes pulmonary vasodilation and bronchodilation [16–18], and both mean arterial pressure and heart rate increase. Its profile on systemic hemodynamics is favorable and its depressant effects on the gastrointestinal system are minimal; finally, Ketamine can have also antiplatelets activity by inhibiting phosphoinositide breakdown and intracellular Ca^2+^ mobilization in human platelets stimulated by collagen [16–18].

## Which are the cerebral effects of ketamine?

The physiological mechanisms leading to neuroprotection by ketamine are depicted in Additional file 3: Figure S3. Ketamine causes vasodilation and increased cerebral blood flow [5, 25]. The metabolic rate of oxygen also increases, although in a heterogeneous way, increasing in the frontal lobes and the insula, while decreasing in the temporal lobes, the cerebellum or pons [11, 16, 25]. Cerebral blood flow does not follow the same pattern, possibly due to dose-dependent uncoupling mechanism [11, 16, 25]. Intracranial pressure remains unchanged or decreases associated with increases in cerebral perfusion pressure [11, 16, 18, 25]. Cerebral oxygenation remains unchanged [26, 27]. Moreover, ketamine does not compromise the autoregulatory mechanisms or the carbon dioxide (CO_2_) reactivity of the cerebral vasculature [25]. Additionally, it is able to decrease seizures and non-convulsive epileptic activity [11, 16–18] -and has neuroprotective effects [28–33] by reducing glutamate levels and inhibiting cortical spreading depressive depolarization.

Despite the fact that glutamate plays a role in the mechanisms of damage in the initial phases post TBI and stroke (excitotoxicity), clinical studies employing “anti-NMDA” agents have consistently failed in improving outcomes [34]. The hypotheses that attempt to explain these results consists in the fact that glutamate fulfills a dual function: immediately after the injury it acts as a mediator of toxicity, and then resumes its physiological function promoting phenomena that promote neuronal repair and survival [34]. On the other hand, it is important to highlight that NMDA receptors have different protein subpopulations in their composition, capable of triggering different pathways that contribute to proliferation, neuronal regeneration or synaptogenesis, depending on which protein is stimulated [35].

Pre-clinical and clinical studies have shown that after TBI or stroke, NMDA receptors remain hypofunctional and this could be associated with cognitive deficits. Activating these receptors by alternative routes (glycine/serine) is a promising strategy [36].

## What is the available clinical evidence?

For decades, the use of ketamine was precluded in TBI patients based on evidence from the 70 s that suggested its detrimental effect on intracranial pressure [37]. Reports in healthy volunteers or in individuals without neurological pathology undergoing general surgical procedures, showed that intracranial pressure, cerebral blood flow and cerebral perfusion pressure increase while anesthesia was induced with variable doses of ketamine but no neurological complications or sequels were reported [38, 39]. Uncontrolled series with small numbers of patients in different central nervous system pathologies that had in common abnormal cerebral spinal fluid circulation showed similar results, highlighting the absence of adverse consequences [40–45]. More recent systematic reviews with different degrees and types of limitations conclude in heterogeneous acute brain populations (subarachnoid hemorrhage, tumors, TBI), that ketamine causes only temporary changes in intracranial pressure without modifying cerebral perfusion pressure, and has no detrimental effect on intensive care unit stay, outcomes, or mortality [46–48] When analyzing populations of severe TBI, ketamine was found not to increase intracranial pressure in sedated and normocapnic mechanically ventilated patients (Oxford level 2b, Grade C); moreover, ketamine may decrease intracranial pressure in selected cases [49]. Recent update of ketamine use in acute brain injury lead to similar conclusions [50] (Table 1).

**Table 1:** Ketamine in severe TBI

Author			Year	Population	Number	Study	Doses	ICP response	CPP response	Adverse effects
Bar Joseph et al.			2009	Adult Mechanical ventilated Severe TBI	*N* = 25	Prospective Group A: normal ICP Group B: high ICP	Bolus 1–1.5 mg/kg	Group A: No increase Group B: Decrease 32% (Mean 25.7 to 17.6 mmHg)	NA	None
Bar Joseph et al.			2009	Pediatric mechanical ventilated severe TBI	*N* = 30	Prospective Group 1: ketamine prevention Group B: ketamine to reduce ICP	Bolus 1–1.5 mg/kg	Group A: ICP decrease (Mean 25.2 to 17.9 mmHg) Group B: ICP decrease (Mean 26 to 17.5 mmHg)	Group A and Group B: CPP increase	None
Boirgoin et al.			2005	Adult mechanical ventilated severe TBI	*N* = 30	Prospective Group 1: sufentanyl + Midazolam Group 2: ketamine + midazolam	Continuous infusión to achieve plasma concentration of 1 mcg/ml	No difference in ICP ICP non increase (non reported values)	No difference	None
Boirgoin et al.			2003	Adult mechanical ventilated severe TBI	*N* = 25	Prospective Group 1: ketamine + Midazolam Group 2: Midazolam + sufentanyl	Continuous infusión 82 mcg/kg/min	No difference between groups (Mean 19 vs 15.7 mmHg-p = 0.28)	NA	Non significant increase in HR
Albanese et al.			1997	Adult mechanical ventilated severe TBI	*N* = 8	Prospective	Bolus 1.5; 3; and 5 mg/kg	ICP decrease from baseline at all doses (Mean 2, 4 and 5 mmHg at 1,5; 3 and 5 mg/kg, respectively)	NA	None
Kolenda et al.			1996	Adult mechanical ventilated moderate and severe TBI	*N* = 24	Prospective Group 1: ketamine + Midazolam Group 2: fentanyl + midazolam	Continuous infusión 104 mg/kg/day	ICP 2 mmHg higher in ketamine group only at day 8 of infusion	CPP 8 mmHg higher in ketamine group	None
Schmitter et al.			2007	Adult mechanical ventilated severe TBI	*N* = 24	Prospective Group 1: fentanyl + methohexitone Group 2: ketamine + methohexitone	Continuous infusión 2 mg/kg/h	No difference between groups Mean 15.9 vs 14.7 mmHg)	No difference between groups	None

## Safety profile

At recommended doses, Ketamine is a safe drug [16, 17, 50]. The most common reported side effects are nausea, vomiting, dizziness, diplopia, drowsiness, dysphoria, and confusion. Hallucinations are generally minimized by the concomitant use of benzodiazepines and propofol. Delirium rates oscillate between 6 and 12%. There are no studies that have assessed undesirable effects after long-term infusion. Systematic reviews emphasize the absence of severe or fatal adverse effects [48–50].

## Is ketamine cost-effective?

Ketamine is not an expensive drug. There are no studies that have evaluated the cost effectiveness of ketamine in the management of sedation and analgesia of patients with severe TBI; however, the beneficial properties of ketamine on hemodynamic and respiratory status could potentially allow less concomitant use of fluids, vasopressors, and perhaps fewer days of mechanical ventilation [7, 8, 11, 16–18]. A 50 mg/ml vial of ketamine costs 4.4 euros in comparison to 20 mg/ml vial of propofol whose cost is 10.77 euros. Official data from the Spanish Medicines Agency (www.aemps.gob.es) presenting the daily cost of a continuous infusion of standard doses of sedatives and analgesics in a 70 kg individual are shown in Additional file 4: Table S1.

## When not to use ketamine?

First, when there is hypersensitivity to the agent [50]. Due to its hepatic metabolism and almost exclusive renal elimination, its use is not recommended in the context of kidney and/or liver failure [16, 17, 50]. It should also not be used in situations where high blood pressure triggers potentially dangerous complications such as diastolic cardiac dysfunction, acute myocardial infarction, or aortic dissection [37, 50].

Increased toxicity of ketamine has been reported in the settings of severe alcoholism [50]. Moreover, in severe TBI, certain situations would contraindicate its use, such as loss of cerebral autoregulation, hydrocephalus, or the concomitant presence of untreated brain aneurysms [16, 17, 50]. Finally, ketamine may have psychotomimetic effects which could be the main reason for not using ketamine as a primary agent in the ICU [5, 6].

## When to use ketamine

Drug choices should take in account airway management, sedation, analgesia and hemodynamic integrity, as many sedatives may cause transient hypotension and/or apnea. Due to its pharmacokinetic and pharmacodynamic properties and its systemic and central nervous system effects, ketamine is an attractive option to consider in the management of severe TBI under mechanical ventilation in normocapnic condition and associated with other sedatives agents (propofol, midazolam). Moreover, even in moderate TBI ketamine can have a role especially for the management of agitation with consequent risk of secondary insults, but at the same time to allow neurological assessment and early detect neuroworsening, while maintaining hemodynamic stability and without depressing respiratory function.

Certain situations may favor its employment in TBI (Additional file 5: Figure S4):

- Prehospital setting. In the acute phase of resuscitation, especially in low-resource regions, ketamine can be an interesting option because it potentially helps to maintain a stable hemodynamic profile, avoiding hypotension, reducing the use of fluids and maintaining spontaneous ventilation.
- Hemodynamic unstability. Its sympathomimetic properties help to maintain a more stable hemodynamic profile in different associated situations, such as shock from any etiology [7, 8, 11, 16–18].
- Airway resistance increase. Ketamine is an option to be considered when severe TBI is associated with asthma or chronic obstructive pulmonary disease (COPD) or another situations that cause severe bronchospasm [7, 8, 11, 16–18].
- Refractory convulsive, non-convulsive status, or suspect of spreading depolarization [7, 8, 11, 16–18].
- Need for non-opioid analgesia [16, 17].
- Conscious sedation. In this sedation modality, it is necessary to be able to neurologically evaluate the patient while maintaining spontaneous ventilation and stable hemodynamics, such as during the weaning process.
- Gastrointestinal dysfunction (gastroparesis, ileus, nutrition intolerance). Ketamine, unlike opioids agents, does not interfere with gastrointestinal motility, a multifactorial and frequent condition in severe TBI.
- Withdrawal syndromes. Its beneficial systemic and cerebral effects make it an option to consider in such situations [16, 17].

## Conclusions

In conclusion, due to its specific pharmacological and pharmacokinetics characteristics, including neuromodulation properties, ketamine might be considered as a potential drug to optimize analgesia and sedation in the management of mechanically ventilated patients with severe TBI in association with other sedatives agents. Further, the versatility and safety profile of ketamine makes it a reliable tool in the prehospital environment. Adequately powered, randomized controlled trials and new guidelines should aim to refining the role and place of ketamine in patients with traumatic brain injury.

## Supplementary information

**Additional file 1: Figure S1.** Systemic and cerebral consequences of pain and agitation. SIRS: systemic inflammatory response syndrome; Tº: central temperature; HR: heart rate; pa02: oxygen arterial pressure; paCO2: carbon dioxide arterial pressure; coagul: coagulation alterations; IAP: intraabdominal pressure; UO: urinary output; Hypovol: hypovolemia; Hypergly: hyperglycemia; ICP: intracranial pressure; CBF: cerebral blood flow; CPP: cerebral perfusion pressure; CMO2: cerebral metabolic rate of oxygen; CMGl: cerebral metabolic rate of glycemia; CBV: cerebral blood volume.

**Additional file 2: Figure S2**. Sedation and Analgesia ideal level.

**Additional file 3: Figure S3.** Mechanisms of neuroprotection by ketamine. Ketamine is a “glutamate modulator.” It exerts its effect basically at two levels: a) presynaptic, inhibiting the release of glutamate and b) post-synaptic, acting as a competitive blocker of N-Methyl-D-Aspartate receptors, thereby excitotoxic injury through inhibiting the entry of calcium into cells, the formation of nitric oxide and oxygen free radicals. Modulates glucose metabolism and the generation of mitochondrial ATP. Inhibits the apoptotic phenomenon. Additionally, it inhibits spreading depolarizations and acts as an antithrombotic and anti-inflammatory by inhibiting platelet aggregation and the production and release of cytokines by the microglia. IL-8: interleukin-8; TNF: tumor necrosis factor; Ca^++^: calcium; K^+^: potassium; OFR: oxygen free radicals; ATP: adenosine triphosphate; CBF: cerebral blood flow.

**Additional file 4: Table S1.** Comparative cost of 24 hours intravenous infusion of sedative and analgesic drugs. mg: milligrams; kg: kilograms; h: hour; min: minute; mcg: micrograms

**Additional file 5: Figure S5.** Potential indications of ketamine continuous infusion in traumatic brain injury.

## Data Availability

Not applicable.

## References

[1] Maas AI, Stocchetti N, Bullock R (2008). Moderate and severe traumatic brain injury in adults. Lancet Neurol.

[2] Godoy DA, Lubillo S, Rabinstein AA (2018). Pathophysiology and management of intracranial hypertension and tissular brain hypoxia after severe traumatic brain injury: an integrative approach. Neurosurg Clin N Am.

[3] Devlin JW, Skrobik Y, Gélinas C (2018). Clinical practice guidelines for the prevention and management of pain, agitation/sedation, delirium, immobility, and sleep disruption in adult patients in the ICU. Crit Care Med.

[4] Carney N, Totten AM, O’Reilly C, Ullman JS, Hawryluk GW, Bell MJ (2017). Guidelines for the management of severe traumatic brain injury, fourth edition. Neurosurgery.

[5] Takeshita H, Okuda Y, Sari A (1972). The effects of ketamine on cerebral circulation and metabolism in man. Anesthesiology.

[6] Kolenda H, Gremmelt A, Rading S, Braun U, Markakis E (1996). Ketamine for analgosedative therapy in intensive care treatment of head-injured patients. Acta Neurochir (Wien).

[7] Oddo M, Crippa IM, Metha S, Menon D, Payen JF, Taccone FB, Citerio G (2016). Optimizing sedation in patients with acute brain injury. Crit Care.

[8] Roberts DJ, Hall RI, Kramer AH, Robertson HL, Gallagher CN, Zygun DA (2011). Sedation for critically ill adults with severe traumatic brain injury: a systematic review of randomized controlled trials. Crit Care Med.

[9] Sessler CN, Gosnell MS, Grap MJ, Brophy GM, O’Neal PV, Keane KA (2002). The Richmond Agitation Sedation Scale. Validity and reliability in adult intensive care unit patients. Am J Resp Crit Care Med.

[10] Deogaonkar A (2004). Bispectral Index monitoring correlates with sedation scales in brain-injured patients. Crit Care Med.

[11] Opdenakker O, Vanstraelen A, De Sloovere V, Meyfroidt G (2019). Sedatives in neurocritical care: an update on pharmacological agents and modes of sedation. Curr Opin Crit Care.

[12] Zheng J, Gao Y, Xu X, Kang K, Liu H, Wang H, Yu K (2018). Correlation of bispectral index and Richmond agitation sedation scale for evaluating sedation depth: a retrospective study. J Thorac Dis.

[13] Vincent JL, Takala J, Moreno RP, Sakr Y, Marshall JC (2016). The Richmond Agitation–-Sedation scale should not be used to evaluate neurologic function. Crit Care Med.

[14] Akeju O, Song AH, Hamilos AE (2016). Electroencephalogram signatures of ketamine anesthesia-induced unconsciousness. Clin Neurophysiol.

[15] Vlisides PE, Bel-Bahar T, Lee U, Li D, Kim H, Janke E, Tarnal V, Pichurko AB, McKinney AM, Kunkler BS, Picton P, Mashour GA (2017). Neurophysiologic correlates of ketamine sedation and anesthesia: a high-density electroencephalography study in healthy volunteers. Anesthesiology.

[16] Himmelseher S, Durieux ME (2005). Revising a dogma: ketamine for patients with neurological injury?. Anesth Analg.

[17] Hurth KP, Jaworski A, Thomas KB, Kirsch WB, Rudoni MA, Wohlfarth KM (2020). The reemergence of ketamine for treatment in critically ill adults. Crit Care Med.

[18] Flower O, Hellings S (2012). Sedation in traumatic brain injury. Emerg Med Int.

[19] Zanos P, Moaddel R, Morris PJ (2018). Ketamine and ketamine metabolite pharmacology: insights into therapeutic mechanisms [published correction appears in Pharmacol Rev. 2018 Oct;70(4):879]. Pharmacol Rev.

[20] Mion G, Villevieille T (2013). Ketamine pharmacology: an update (pharmacodynamics and molecular aspects, recent findings). CNS Neurosci Ther.

[21] Kamp J, Olofsen E, Henthorn TK, van Velzen M, Niesters M, Dahan A (2020). Ketamine pharmacokinetic study group. Ketamine Pharmacokinet Anesthesiol.

[22] Hartings JA (2020). Prognostic value of spreading depolarizations in patients with severe traumatic brain injury. JAMA Neurol.

[23] Sánchez-Porras R (2017). Ketamine modulation of the haemodynamic response to spreading depolarization in the gyrencephalic swine brain. J Cereb Blood Flow Metab.

[24] Reinhart KM, Shuttleworth CW (2018). Ketamine reduces deleterious consequences of spreading depolarizations. Exp Neurol.

[25] Slupe AM, Kirsch JR (2018). Effects of anesthesia on cerebral blood flow, metabolism, and neuroprotection. J Cereb Blood Flow Metab.

[26] Albanese J, Arnaud S, Rey M, Thomachot L (1997). Ketamine decreases intracranial pressure and electroencephalographic activity in traumatic brain injury patients during propofol sedation. Anesthesiology.

[27] Bhaire VS, Panda N, Luthra A, Chauhan R, Rajappa D, Bhagat H (2019). Effect of combination of ketamine and propofol (ketofol) on cerebral oxygenation in neurosurgical patients: a randomized double-blinded controlled trial. Anesth Essays Res.

[28] Sakai T, Ichiyama T, Whitten CW (2000). Ketamine suppresses endotoxin-induced NF-kappaB expression. Can J Anaesth.

[29] Bhutta AT, Schmitz ML, Swearingen C (2012). Ketamine as a neuroprotective and anti-inflammatory agent in children undergoing surgery on cardiopulmonary bypass: a pilot randomized, double-blind, placebo-controlled trial. Pediatr Crit Care Med.

[30] Carlson AP, Abbas M, Alunday RL, et al. Spreading depolarization in acute brain injury inhibited by ketamine: a prospective, randomized, multiple cross-over trial. J Neurosurg 2018;1–7. 10.3171/2017.12.JNS171665 PMC6279620 29799344

[31] Pfenninger E, Himmelseher S (1997). Neuroprotektion durch Ketamin auf zellulärer Ebene [Neuroprotection by ketamine at the cellular level]. Anaesthesist.

[32] Hudetz J, Pagel P (2009). Neuroprotection by ketamine: a review of the experimental and clinical evidence. J Cardiothor Vasc Anesth.

[33] Bell JD (2017). In Vogue: ketamine for neuroprotection in acute neurologic injury. Anesth Analg.

[34] Ikonomidou C, Turski L (2002). Why did NMDA receptor antagonists fail clinical trials for stroke and traumatic brain injury?. Lancet Neurol.

[35] Lai TW (2014). Excitotoxicity and stroke: identifying novel targets for neuroprotection. Prog Neurobiol.

[36] Biegon A (2018). Stimulation of N-methyl-d-aspartate receptors by exogenous and endogenous ligands improves outcome of brain injury. Curr Opin Neurol.

[37] Green SM, Andolfatto G, Krauss BS (2015). Ketamine and intracranial pressure: no contraindication except hydrocephalus. Ann Emerg Med.

[38] Sari A, Okuda Y, Takeshita H (1972). The effect of ketamine on cerebrospinal fluid pressure. Anesth Analg.

[39] Gardner AE, Olson BE, Lichtiger M (1971). Cerebrospinal-fluid pressure during dissociative anesthesia with ketamine. Anesthesiology.

[40] Tjaden RJ, Ethier R, Gilbert RG (1969). The use of CI-581 (Ketalar) for pediatric pneumoencephalography. J Can Assoc Radiol.

[41] Evans J, Rosen M, Weeks RD (1971). Ketamine in neurosurgical procedures [letter]. Lancet.

[42] Gibbs JM (1972). The effect of intravenous ketamine on cerebrospinal fluid pressure. Br J Anaesth.

[43] List WF, Crumrine RS, Cascorbi HF (1972). Increased cerebrospinal fluid pressure after ketamine. Anesthesiology.

[44] Lockhart CH, Jenkins JJ (1972). Ketamine induced apnea in patients with increased intracranial pressure. Anesthesiology.

[45] Shapiro HM, Wyte SR, Harris AB (1972). Ketamine anaesthesia in patients with intracranial pathology. Br J Anaesth.

[46] Cohen L, Athaide V, Wickham M (2015). The effect of ketamine on intracranial and cerebral perfusion pressure and health outcomes: a systematic review. Ann Emerg Med.

[47] Loflin R, Koyfman A (2015). When used for sedation, does ketamine increase intracerebral pressure more than fentanyl or sufentanil?. Ann Emerg Med.

[48] Wang X, Ding X, Tong Y (2014). Ketamine does not increase intracranial pressure compared with opioids: meta-analysis of randomized controlled trials. J Anesth.

[49] Zeiler FA, Teitelbaum J, West M, Gillman LM (2014). The ketamine effect on ICP in traumatic brain injury. Neurocrit Care.

[50] Gregers MCT, Mikkelsen S, Lindvig KP, Brøchner AC (2020). Ketamine as an anesthetic for patients with acute brain injury: a systematic review [published online ahead of print, 2020 Apr 23]. Neurocrit Care.

