# Supplementary figures and images for: Ketamine in acute phase of severe traumatic brain injury “an old drug for new uses?”
Source: Crit Care. 2021 Jan 6;25:19. doi: 10.1186/s13054-020-03452-x (PMC7788834; doi:10.1186/s13054-020-03452-x)

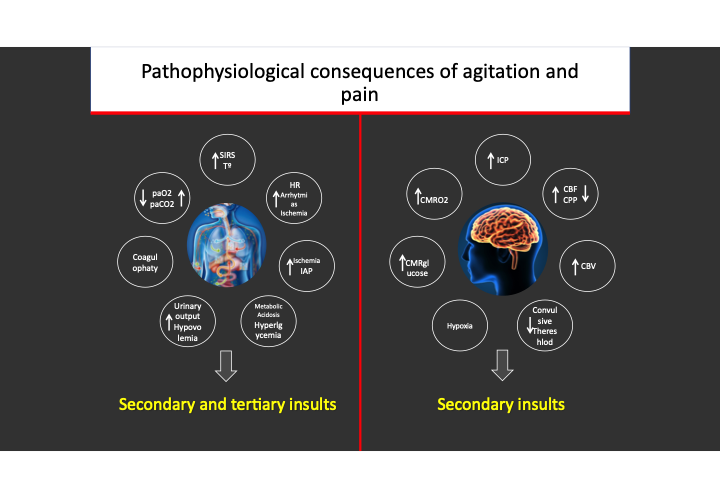

Supplement: Supplementary file 1 — Additional file 1: Figure S1. Systemic and cerebral consequences of pain and agitation. SIRS: systemic inflammatory response syndrome; Tº: central temperature; HR: heart rate; pa02: oxygen arterial pressure; paCO2: carbon dioxide arterial pressure; coagul: coagulation alterations; IAP: intraabdominal pressure; UO: urinary output; Hypovol: hypovolemia; Hypergly: hyperglycemia; ICP: intracranial pressure; CBF: cerebral blood flow; CPP: cerebral perfusion pressure; CMO2: cerebral metabolic rate of oxygen; CMGl: cerebral metabolic rate of glycemia; CBV: cerebral blood volume. [file 13054_2020_3452_MOESM1_ESM.tiff]

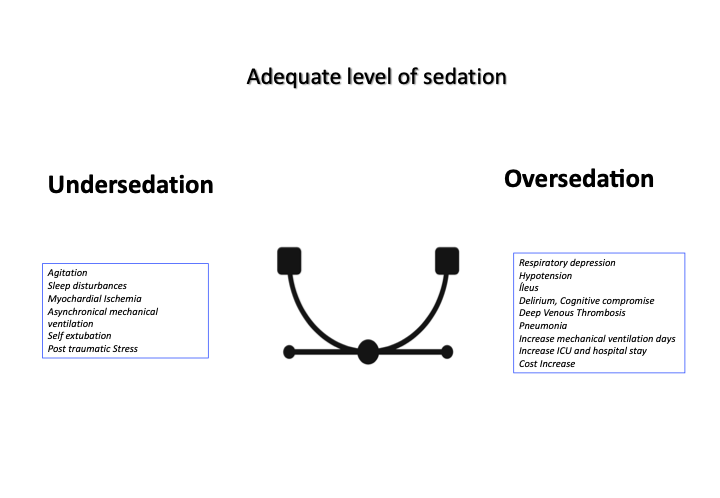

Supplement: Supplementary file 2 — Additional file 2: Figure S2. Sedation and Analgesia ideal level. [file 13054_2020_3452_MOESM2_ESM.tiff]

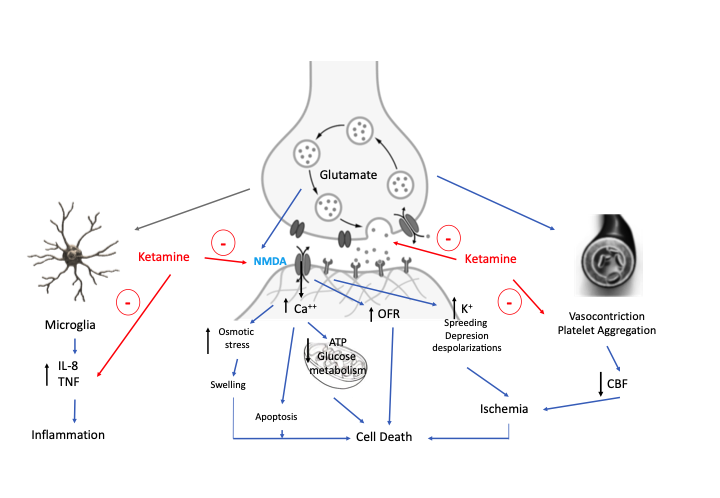

Supplement: Supplementary file 3 — Additional file 3: Figure S3. Mechanisms of neuroprotection by ketamine. Ketamine is a “glutamate modulator.” It exerts its effect basically at two levels: a) presynaptic, inhibiting the release of glutamate and b) post-synaptic, acting as a competitive blocker of N-Methyl-D-Aspartate receptors, thereby excitotoxic injury through inhibiting the entry of calcium into cells, the formation of nitric oxide and oxygen free radicals. Modulates glucose metabolism and the generation of mitochondrial ATP. Inhibits the apoptotic phenomenon. Additionally, it inhibits spreading depolarizations and acts as an antithrombotic and anti-inflammatory by inhibiting platelet aggregation and the production and release of cytokines by the microglia. IL-8: interleukin-8; TNF: tumor necrosis factor; Ca++: calcium; K+: potassium; OFR: oxygen free radicals; ATP: adenosine triphosphate; CBF: cerebral blood flow. [file 13054_2020_3452_MOESM3_ESM.tiff]
