# Supplementary material for: Ketamine in acute phase of severe traumatic brain injury “an old drug for new uses?”
Source: Crit Care. 2021 Jan 6;25:19. doi: 10.1186/s13054-020-03452-x (PMC7788834; doi:10.1186/s13054-020-03452-x)
Supplement: Supplementary file 4 — Additional file 4: Table S1. Comparative cost of 24 hours intravenous infusion of sedative and analgesic drugs. mg: milligrams; kg: kilograms; h: hour; min: minute; mcg: micrograms [file 13054_2020_3452_MOESM4_ESM.docx]

Table S1

| Drug | Dose | Daily cost (euros) |
| --- | --- | --- |
| Midazolam | 0.2 mg/kg/h | 90 € |
| Propofol | 2 mg/kg/h | 361 € |
| Ketamine | 1 mg/kg/h | 147 € |
| Remifentanyl | 0.1 mcg/kg/min | 1200 € |
| Fentanyl | 0.7 mcg/kg/h | 312 € |
| Dexmedetomidine | 0.2 mcg/kg/h | 2100 € |
